# Dr. Vivian Poore's Views

**Published:** 1902-10-18

**Authors:** 


### DR. VIVIAN POORE'S VIEWS.

To show that this question as to the proper manner of disposing of excreta is not yet finally settled, and that, to some minds, the knowledge that it is possible to sterilise them does not of necessity carry with it the obligation to do so, we may refer to Dr. Vivian Poore's well-known views. Organic refuse, he says, is always the soldier's deadliest enemy, but instead of attempting to sterilise it, a task the magnitude of which is perhaps hardly appreciated by some writers, he would still have them buried in the soil, and to show that this earth to earth method, if properly carried out, is not a means of disseminating infection he instances the great farm on Carrington Moss, where, during the past thirteen years, 639,779 tons of refuse, much of it pail refuse, has been utilised, and on which farm during the past sixteen years there has been but one case of enteric fever, and that one in a young Irish labourer who had recently arrived. The use of tubs and dry-earth systems in towns is of course another affair, all that Dr. Poore refers to is the alleged danger of the practice of applying the undisinfected excreta to the soil. Evidently the question is not yet finally answered.

